# Cylindrical Dielectric Resonator Antenna-Based Sensors for Liquid Chemical Detection

**DOI:** 10.3390/s19051200

**Published:** 2019-03-08

**Authors:** Amjad Iqbal, Amor Smida, Omar A. Saraereh, Qais H. Alsafasfeh, Nazih Khaddaj Mallat, Byung Moo Lee

**Affiliations:** 1 Centre for Wireless Technology (CWT), Faculty of Engineering, Multimedia University, Cyberjaya 63100, Malaysia; 2 Department of Medical Equipment Technology, College of Applied Medical Sciences, Majmaah University, AlMajmaah 11952, Saudi Arabia; a.smida@mu.edu.sa; 3 Unit of Research in High Frequency Electronic Circuits and Systems, Faculty of Mathematical, Physical and Natural Sciences of Tunis, Tunis El Manar University, Tunis 2092, Tunisia; 4 Department of Electrical Engineering, Hashemite University, Zarqa 13115, Jordan; eloas2@hu.edu.jo; 5 Department of Electrical Power and Mechatronics Engineering, Tafila Technical University, Tafila 11183, Jordan; qsafasfeh@ttu.edu.jo; 6 College of Engineering, Al Ain University of Science and Technology, Al Ain 64141, UAE; nazih.mallat@aau.ac.ae; 7 School of Intelligent Mechatronics Engineering, Sejong University, Seoul 05006, Korea

**Keywords:** dielectric resonator, chemical sensing, S-parameters, circuit model

## Abstract

A compact, cylindrical dielectric resonator antenna (CDRA), using radio frequency signals to identify different liquids is proposed in this paper. The proposed CDRA sensor is excited by a rectangular slot through a 3-mm-wide microstrip line. The rectangular slot has been used to excite the CDRA for $HEM_{11}$ mode at 5.25 GHz. Circuit model values (capacitance, inductance, resistance and transformer ratios) of the proposed CDRA are derived to show the true behaviour of the system. The proposed CDRA acts as a sensor due to the fact that different liquids have different dielectric permittivities and, hence, will be having different resonance frequencies. Two different types of CDRA sensors are designed and experimentally validated with four different liquids (Isopropyl, ethanol, methanol and water).

## 1. Introduction

Chemical sensors have been utilized for a long time to distinguish the purity/impurity of several liquids/chemicals to improve the course of action of those liquids/chemicals for an extensive variety of modern applications. It is important to store the liquids/chemicals as per the Globally Harmonized System of Classification and labelling of liquids/chemicals. The utilization of nameless liquids/chemicals may lead to unexpected results including harming human organs. Thus, liquids/chemicals ought to be marked with the goal that they are perceived appropriately [1].

Material characterization and sensing have recently gained much more attention due to the fast developing interest in various industries. Each material has a distinctive behaviour, based on the properties and nature of the dielectric material in the presence of the electromagnetic fields. The nature and behaviour of the dielectric material are determined by analyzing properties such as permittivity, permeability and conductivity. Permittivity is a vital characteristic among all the properties, based on which the electrical nature of the material is determined. Various types of sensors and sensor combinations are utilized for chemical sensing and material characterization in different industries; however, these methodologies have some disadvantages like high power consumption, large size, complicated design and uneconomical usage [2]. Radiofrequency (RF) and microwave sensing for chemical identification and material characterization is considered one of the best sensing techniques because of its cost-effectiveness, low power consumption and easy fabrication [3,4,5].

Formerly, the techniques used for looking at bioassays and evaluating fluid quality required enormous volumes of fluids/chemicals to fill the testing tubes for analysis [6,7,8]. An extensive amount of those fluids remain unused, disposed of and never utilized for exploratory necessities. For eliminating this issue of squandering unused fluidic chemicals, microfluidic courses of action were declared. Investigation of the fluids/chemicals would now be able to be accomplished on completely little volumes, typically at the micro-liter or nano-litre range of the fluids/chemicals. Liquid/chemical sensing technology has gained much more advancement through the development of chip-based compact waveguides [9,10,11]. A high-quality factor, SIW resonator, having confined energies at the centre, is utilized as a humidity-sensing sensor [8]. A gas detection sensor is designed using a multi-mode (Specifically $TE_{101}$ and $TE_{102}$) SIW resonator [12]. A complementary split ring resonator-based sensor for liquid dielectric characterization is reported in [13], in which the material under testing is placed in a normal orientation to the sensor surface.

Several attempts for performance enhancement have been carried out while keeping the sensor as small as possible. Several Substrate Integrated Waveguide (SIW) based sensors were designed for different sensing applications. Two unequal microfluidic channels are placed in highly intensive electric field regions in the SIW cavity for dual chemical detection [14]. Circular SIW cavity is utilized for single chemical detection [15]. Dual mode resonator utilizing a folded microstrip line is utilized for dual chemical sensing [16]. A number of studies have been carried out for ethanol liquid sensing using SIW [17,18,19]. An inject-printed microstrip patch antenna is used as a sensor for liquid identification in [20].

In this paper, a cylindrical dielectric resonator antenna-based sensor is proposed and experimentally validated for different chemicals (Isopropyl, ethanol, methanol and water). The proposed DRA-based sensors shows a reasonable sensing capability as compared to the existing sensing technologies. Main contributions of the proposed work are given below

- To the best of our knowledge, this is the first ever DRA-based constructed sensor for chemical liquid detection.
- A minimal size, large frequency shift and high sensitivity make it a good candidate to be used for chemical sensing.

## 2. Design Methodology

### 2.1. Dielectric Resonator Antenna Characterization

**Figure 1:**
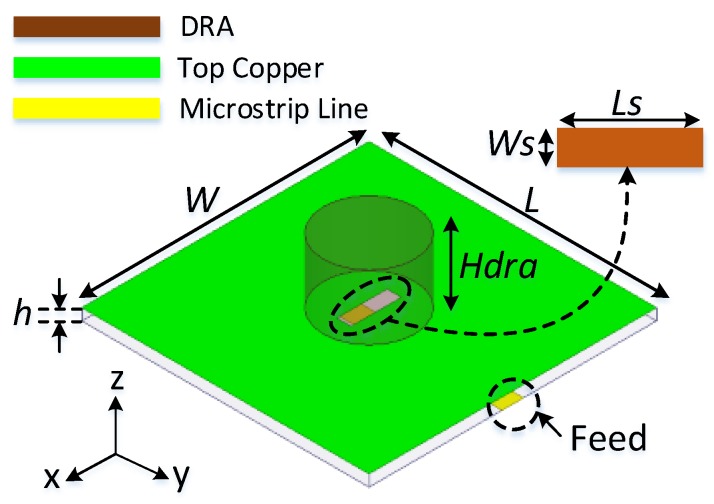
Proposed cylindrical dielectric resonator antenna to be used as a sensor (*Ls* = 7 mm, *Ws* = 2 mm, *Hdra* = 9 mm, *h* = 1.6 mm).

Figure 1 shows the configuration of the proposed cylindrical dielectric resonator antenna. The radiating element (CDRA) is placed on a 1.6 mm thicker FR-4 substrate with a relative permittivity of 4.4 and a loss tangent of 0.019. The width and length of the substrate are chosen as 30 mm. TMM 10i material having a relative permittivity of 9.8 and loss tangent of 0.002 is utilized for designing the CDRA. The CDRA has a height of 9 mm and a radius of 6.35 mm. To feed the antenna, a 50 Ω microstrip line is used. A rectangular slot below the CDRA is designed to feed the CDRA. The proposed DRA resonates at 5.25 GHz with 10 dB bandwidth of 340 MHz (5.08–5.42 GHz) covering most of the application bands such as Mi-WiFi, WLAN and ISM band. the proposed antenna have high gain of 7.1 dBi and efficiency of 82%. The proposed DRA can be used in applications such as Mi-WiFi, WLAN and ISM band because of its high quality factor, good impedance matching, high gain and efficiency. Also, the compact structure of the antenna makes it a suitable choice for system-in-package (SIP) applications.

The resonance frequency of a rectangular-shaped slot for fundamental ($TM_{10}$) mode can be calculated as follows [21].

(1) $$f_{r}=\frac{c}{2L_{s}}\sqrt{\frac{2}{1+\epsilon_{s}}}$$

where *c* shows the speed of light and $\epsilon_{s}$ represents permittivity of the substrate which is 4.4. From (1), the resonance frequency of a rectangular shaped slot for fundamental ($TM_{10}$) mode is found to be 5.1 GHz.

In the same way, the resonance frequency of $HEM_{11}$ mode in CDRA can be calculated as follow [22].

(2) $$f_{r}=\frac{6.321c}{\pi D\sqrt{\epsilon_{r,eff}+2}}\left[0.27+0.36\left(\frac{2r}{4H_{eff}}\right)+0.02{\left(\frac{2r}{4H_{eff}}\right)}^{2}\right]$$

where *r* represents the radius of the CDRA, $H_{eff}$ represents the effective height of the resonator. $H_{eff}$ is the sum of the height of the CDRA and substrate thickness (h+hdra). $\epsilon_{r,eff}$ is the effective permittivity of the proposed CDRA and can be calculated as [22].

(3) $$\epsilon_{r,eff}=\frac{H_{eff}}{\frac{H}{\epsilon_{r,CDRA}}+\frac{H}{\epsilon_{r,sub}}}$$

#### Excitation Mechanism and Equivalent Circuit Model for CDRA

A rectangular slot of width = 2 mm and length = 7 mm is used for the excitation of the CDRA. The length and width of the rectangular slots are adjusted so as to excite the CDRA for $HEM_{11}$ mode. The radiation resistance of the CDRA depends on the dimensions of the rectangular excitation slot [23].

**Figure 2:**
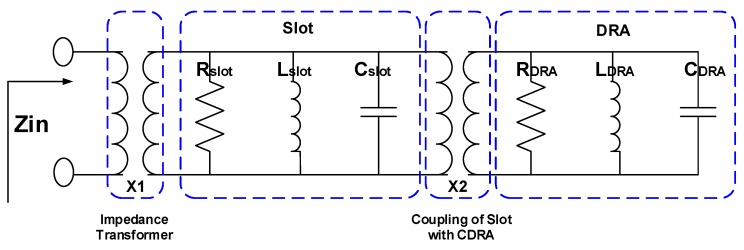
Equivalent circuit model of the complete antenna system.

**Figure 3:**
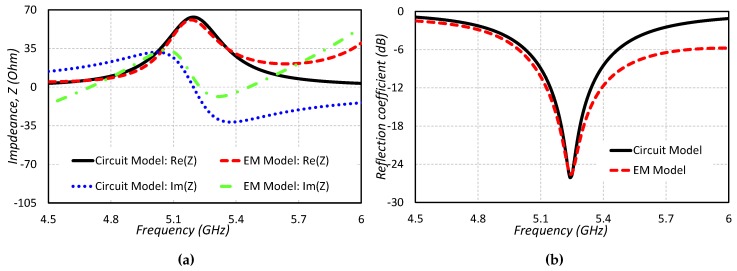
Comparison of the EM model and equivalent circuit model: (**a**) Impedance (**b**) Return loss.

**Figure 4:**
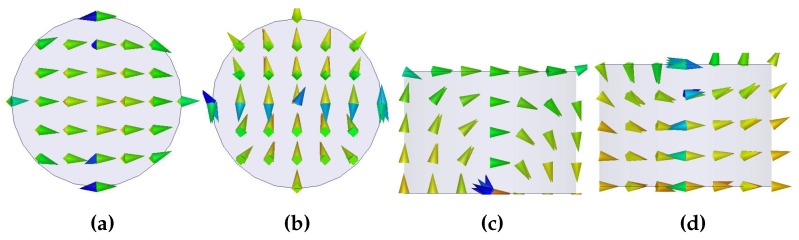
E-fields and H-fields distribution on DRA (**a**) Top view: E-fields (**b**) Top view: H-fields (**c**) Side view: E-fields (**d**) Side view: H-fields.

The equivalent circuit model for the proposed CDRA along with the transmission line and a rectangular slot are shown in Figure 2. In the equivalent circuit model, the impedance transformer represents the 50 Ω transmission line which excites the rectangular slot. Rectangular slot as well as cylindrical DRA is represented with the RLC resonator. The rectangular slot is responsible for installing the $HEM_{11}$ mode in the CDRA. In order to see the validity of the circuit model, the impedance and return loss plots are compared with the EM model. A decent agreement between the real part, imaginary part of the impedance and return loss of the circuit model and EM model is obvious from Figure 3. The optimized values for circuit model ($X_{1}$, $X_{2}$, $R_{slot}$, $L_{slot}$, $C_{slot}$, $R_{DRA}$, $C_{DRA}$ and $L_{DRA}$) are listed in Table 1. The E and H fields of $HEM_{11}$ mode excited in the CDRA are shown in Figure 4.

### 2.2. DRA-Based Sensors Design

#### 2.2.1. Type 1 Sensor

**Figure 5:**
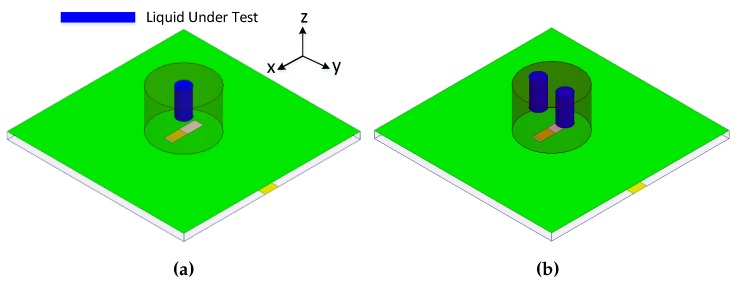
Types of DRA-based sensors (**a**) Type 1: One cylindrical microwell for liquid testing (LT) (**b**) Type 2: Two cylindrical microwells for LT.

**Figure 6:**
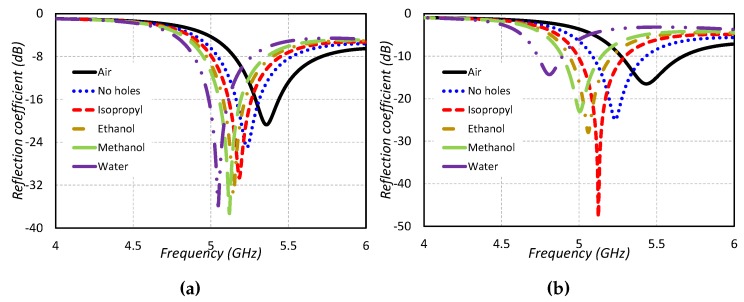
Reflection coefficient of the sensors for different liquid chemicals (**a**) Type 1 sensor (**b**) Type 2 sensor.

The design of this type of sensor is shown in Figure 5a. A cylindrical dielectric resonator antenna is used as a sensing element in this work. A High-Frequency Structure Simulator (HFSS v13.0) is used for designing and optimization purposes. A cylindrical microwell with PDMS is installed in the center of the CDRA for the liquid under test (LUT). A microwell using PDMS values is extracted so as to give proper physical structure in simulations. A large number of simulations have been performed so as to find the optimized radius and height of the microwell basin, keeping in mind the unloaded quality factor of the dielectric resonator as well as the sensing capabilities. It is always desirable for an RF sensor to have a high quality factor so as to intelligently sense dielectric materials of a high dielectric constant value. The optimized microwell basin has the height of 6 mm and radius of 2 mm. The reflection coefficient graphs for the type 1 sensor are given in Figure 6a. Four different types of liquid chemicals (Isopropyl, ethanol, methanol and water) are tested in the designed sensor and their results are shown in Figure 6a. It is clear from Figure 6a that the designed sensor behaves differently for all the liquid chemicals based on their dielectric constant value. The designed sensor is tested under six different conditions: one without PDMS microwell, second with no liquid in the basin, and then testing of four different liquid chemicals. The proposed CDRA has a resonance frequency of 5.25 GHz in the absence of microwell for liquid chemical sensing. The resonance frequency of the CDRA shifts to 5.36 GHz when the microwell is designed at the center of the CDRA. The right hand shift of the resonance frequency of the CDRA is due to the low permittivity of the air inside the microwell. When the microwell is filled with isopropyl, the resonance frequency shifts towards the lower frequency side and noted at 5.16 GHz. The frequency shift of the resonance frequency of CDRA is because of the higher dielectric constant of the isopropyl chemical than the CDRA and air [22]. The resonance frequency of the CDRA sensor is noted at 5.152 GHz, when the ethanol liquid chemicals is tested in the microwell. The resonance frequency shifts to 5.12 GHz for methanol testing. In the case of testing water in the type 1 sensor, the resonance frequency shifts to 5.048 GHz. The large shift of the resonance frequency of type 1 sensor is because of the large dielectric constant value of the water. In this way, the proposed model can be utilized as a sensor using the varying behavior of the resonance frequencies for different liquid chemicals.

Parametric analysis is done in order to discover the average sensitivity for the designed sensor. For this purpose, the loss tangent is kept at 0.4 which is the common value for ethanol at 3–5 GHz and the permittivity is varied. The average sensitivity [24] is determined using the following relation.

$$Average\;Sensitivity\;\left(S1\right)=\frac{\Delta F}{\Delta \epsilon }=8.75\;MHz/\epsilon_{r}$$

**Figure 7:**
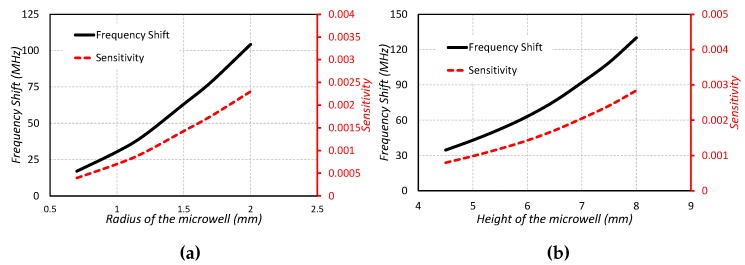
Type1: Frequency shift and sensitivity against (**a**) Radius of the microwell (**b**) Height of the microwell.

The sensing capabilities and frequency shift against the varying radius and height of the microwell is investigated in Figure 7a,b. The following relations are used for determining the fractional change in frequency and the sensitivity [2].

(4) $$Fractional\;change\;in\;Frequency\;\left(F\right)=\frac{f(m)-f(s)}{f(s)}$$

(5) $$Sensitivity=\frac{\Delta F}{\Delta \epsilon^{{}^{\prime }}}$$

where *f(m)* and *f(s)* are the resonance frequencies with and without the liquid under test respectively.

It is noted that the sensitivity and frequency shift values increase with the increase in radius and height of the microwell. The sensitivity and frequency shift of the sensor increase due to the large sample of the liquid chemical as well as more overlap area where the maximum fields exist.

#### 2.2.2. Type 2 Sensor

**Figure 8:**
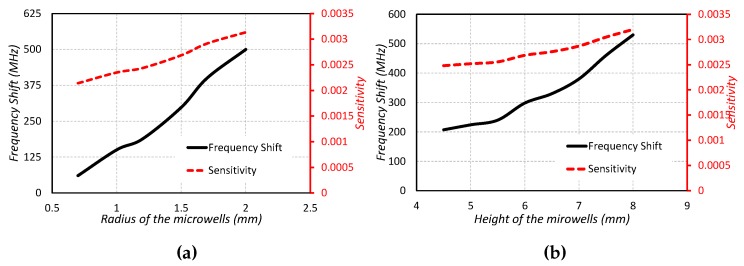
Type2: Frequency shift and sensitivity against (**a**) Radius of the microwells (**b**) Height of the microwells.

This type of DRA-based sensor is shown in Figure 5b. The type 2 sensor is a modified form of the type 1 sensor. Two microwells of the same dimensions for liquid testing are designed in the type 2 sensor. The radius, height and position of the microwell are adjusted so that the sensor has high sensitivity, as well as high quality factor is ensured for sensing the high dielectric constant liquid chemicals. The sensor is tested for four different liquid chemicals and their results are shown in Figure 6b. The average sensitivity is computed as 10.28 MHz/$\epsilon_{r}$. The resonance frequency of the sensor is noted as 5.25 GHz for no microwell in the CDRA. The resonance frequency shifts towards the higher frequency when the two microwells are designed in the CDRA. The resonance frequency is noted as 5.432 GHz for no liquid under test but in the presence of two microwells. The resonance frequency is noted as 5.124 GHz for isopropyl testing. The resonance frequency shifts towards the lower frequency side when the microwells are filled with ethanol chemicals. The resonance frequency in case of ethanol-filled microwells is noted as 5.056 GHz. The resonance frequency further shifts towards the lower frequency side when the methanol liquid is inserted in the microwells. The frequency for methanol-filled microwells is observed as 5.004 GHz. A large frequency shift towards the lower frequency side is obvious from Figure 6b, when the microwells are filled with water. The resonance frequency lies at 4.80 GHz for water-filled microwells. Hence, it is proved that the proposed model can be used as a sensor to differentiate different liquids using the resonance frequency. The sensitivity and frequency shifts of the proposed sensor for varying radius and height of the microwells are investigated in Figure 8a,b.

**Figure 9:**
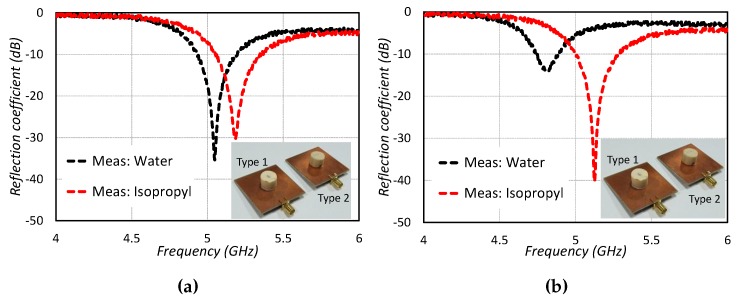
Measured results for CDRA sensor (**a**) Type 1 sensor (**b**) Type 2 sensor.

For measuring the proposed sensors, the reflection coefficient is measured after calibration of the vector network analyzer (VNA). First of all, the reflection coefficient of the sensors without any liquid (empty microwell) is measured by connecting the sensors one by one with VNA. After that, various liquid chemicals are injected into the microwells for chemical sensing. After each measurement, the PDMS microwell is cleaned by exerting air through a pressure syringe and reinstating the reference frequency originating from the empty PDMS microwell. Much consideration is required for the cleaning process and reinstating the reference frequency because contamination may cause a high degree of error in liquid chemical sensing processes. It is noted that all the liquid chemicals tested have different resonance frequencies. Figure 9 shows the measured responses of the proposed sensors. The sensors are fabricated and measured for water and isopropyl.

Table 2 summaries the performance of the two sensors. Table 3 shows the comparative analysis of the proposed sensors with existing RF sensors. It can be well-noted that the proposed sensors have low size, high frequency shift and high sensitivity compared to existing sensors.

## 3. Conclusions

A minimal size cylindrical dielectric resonator antenna (CDRA) using radio frequency signals to identify different liquids is successfully designed, simulated, fabricated and measured in this paper. The rectangular slot has been used to excite the CDRA for $HEM_{11}$ mode at 5.25 GHz. Circuit model values (capacitance, inductance, resistance and transformer ratios) of the proposed CDRA are derived to show the true behaviour of the system. Two different types of sensors are successfully implemented using CDRA. Different resonating frequencies are noted for the different types of chemicals using the two CDRA-based sensors (Type 1 sensor: air = 5.36 GHz, isopropyl = 5.16 GHz, ethano = 5.152 GHz, methanol = 5.12 GHz and water = 5.048 GHz, Type 2 sensor: air = 5.432 GHz, isopropyl = 5.124 GHz, ethanol = 5.056 GHz, methanol = 5.004 GHz and water = 4.80 GHz). An important feature of this sensor is its high quality factor during all chemicals sensing. Also, the sensor can be used as antenna when required due to its high quality factor and good impedance matching at resonance frequency.

## Figures and Tables

**Table 1:** Equivalent circuit model components values.

Components	Values	Components	Values	Components	Values
$R_{slot}$	250 Ω	$R_{DRA}$	950 Ω	$X_{1}$	0.50
$C_{slot}$	0.981 pF	$C_{DRA}$	1.915 pF	$X_{2}$	1.382
$L_{slot}$	0.67 nH	$L_{DRA}$	0.793 nH		

**Table 2:** Comparison of the two types of sensors.

No.	Resonance Frequency	Average Sensitivity ${}^{\;*1}$	$\Delta F{\;}^{*2}$	Sensitivity ${}^{\;*3}$
Type 1 Sensor	5.25 GHz	8.75 MHz/ϵ	202 MHz	0.0024
Type 2 Sensor	5.25 GHz	10.28 MHz/ϵ	450 MHz	0.0031

${}^{*1}$ represents the average sensitivity obtained through relation of $\frac{\Delta F}{\Delta \epsilon }$. ${}^{*2}$ represents the maximum frequency shift among all the chemicals. ${}^{*3}$ is the sensitivity obtained through (5).

**Table 3:** Comparison with the already designed sensors.

Ref.	Size ($\lambda_{g}$)	$f_{0}$ (GHz)	Technology	ΔF (MHz)	Sensing	Sensitivity (MHz/$\epsilon_{r}$)
[8]	0.5 × 0.5	4.4	SIW	500	Single	9.35–101 KHz/RH
[12]	NA	4–5	SIW	112.1, 265.7	Dual	17.5, 25.7
[15]	1.25 × 1.25	5	SIW	380	Single	69.07
[16]	0.21 × 0.21	2.02, 3.34	Microstrip	281, 309	Dual	3.57, 3.9
[17]	NA	4.7	SIW	70	Single	12.73
[18]	0.43 × 0.93	5.85	SIW	38	Single	5.84
[19]	1.7 × 1.98	17	SIW	145	Single	26.36
**Type 1 Sensor**	**0.52 × 0.52**	**5.25**	**DRA**	**202**	**Single**	**8.75**
**Type 2 Sensor**	**0.52 × 0.52**	**5.25**	**DRA**	**450**	**Single**	**10.28**
